# Cytokine Profiles of Head and Neck Squamous Cell Carcinoma Undergoing Dual Immunotherapy With Cetuximab and Pembrolizumab Identify Interferon Gamma-Induced Protein 10 as Novel Biomarker

**DOI:** 10.3389/fonc.2022.795277

**Published:** 2022-02-28

**Authors:** Michael Berszin, Ioannis Michaelides, Julia Siemert, Louisa Röhl, Jana Wellhausen, Theresa Wald, Christopher Bohr, Julian Künzel, Tanja Gradistanac, Andreas Dietz, Veit Zebralla, Markus Pirlich, Susanne Wiegand, Gunnar Wichmann

**Affiliations:** ^1^ Department of Otorhinolaryngology, Head and Neck Surgery, University Hospital Leipzig, Leipzig, Germany; ^2^ Department of Otorhinolaryngology, University Hospital Regensburg, Regensburg, Germany; ^3^ Department of Pathology, University Hospital Leipzig, Leipzig, Germany

**Keywords:** interferon-gamma induced protein 10 (IP-10), CXCL10 (C-X-C motif ligand 10), interferon-gamma (IFN-γ), vascular endothelial growth factor A (VEGF), interleukin 6 (IL-6), head and neck squamous cell carcinoma (HNSCC), *ex vivo* chemo-sensitivity assay, colony formation

## Abstract

**Background:**

Pembrolizumab and cetuximab are antibodies under investigation in head and neck squamous cell carcinoma (HNSCC) either as single agents or combined with cisplatin and other chemotherapeutic drugs, e.g., 5-fluorouracil and/or docetaxel. However, also the combination of both antibodies may have potential in recurrent/metastatic (R/M) HNSCC, in particular in cisplatin-resistant or -refractory cases or patients with comorbid disease, e.g. patients with impaired renal function.

**Methods:**

To clarify potential benefit that may result from such combination, we used the FLAVINO assay, a short-time *ex vivo* assay to compare responsiveness of HNSCC to pembrolizumab, cetuximab and both combined regarding colony formation of epithelial cells of biopsy-derived tumor samples and their cytokine production within three days either without or with stimulation with 10 ng/mL interferon gamma (IFN-γ). Vascular endothelial growth factor A (VEGF), monocyte chemoattractant protein 1 (MCP-1 or CCL2), interleukin 6 (IL-6), IL-8, IFN-γ, and interferon gamma-induced protein 10 (IP-10 or CXCL10) in supernatants were measured by ELISA.

**Results:**

We detected huge heterogeneity in response to cetuximab, pembrolizumab and both combined with and without IFN-γ stimulation. Moreover, we detected a link between IFN-γ induced IP-10 release and improved outcome in those HNSCC patients who were capable to respond to IFN-γ and pembrolizumab, cetuximab and both combined with a further increase in IP-10 production. We derived an “IP-10 score” that independent from clinical characteristics of HNSCC patients and therapy regimens applied was able to predict their outcome.

**Conclusions:**

The heterogeneity in the *ex vivo* response of cetuximab, pembrolizumab and both combined with and without IFN-γ stimulation identifies subgroups of HNSCC patients with deviating OS.

## Introduction

Immunoediting is a dynamic multistep process of interaction between neoplastic cells and the immune system (1). Although the majority of tumor cells are detected and eliminated by innate and adaptive immune cells, some malignant lesions are able to overcome immunosurveillance through immune escape and manifest as clinical diseases (2).

One of the various mechanisms is the utilization of inhibitory immune checkpoints. The expressions of programmed cell death protein ligands 1 and 2 (PD-L1 and PD-L2, respectively) on the surface of malignant cells and their binding to programmed cell death protein 1 (PD-1) on immune cells block immune surveillance. These immune-compromising mechanisms include the maintenance and induction of regulatory T cells (Tregs) (3), transformation of type 1 T helper cells into Tregs (4), and the downregulation of antitumor response (5–7).

Pembrolizumab (Keytruda^®^, MK-3475; Merck Sharp & Dohme Corp., Whitehouse Station, NJ, USA) is a humanized monoclonal anti-PD-1 antibody that disrupts the PD-1:PD-L1/PD-L2 axis. The immune checkpoint inhibitor has led to promising therapy regimens in the treatment of various cancer entities, including head and neck squamous cell carcinoma (HNSCC) (8). Following the promising results of the open-label, multicenter, phase 1b trial KEYNOTE-012, the PD-1 antibody received approval *via* accelerated process for patients with recurrent and/or metastatic HNSCC (R/M HNSCC) as a second-line treatment option after progression on standard platinum-based therapy (9, 10). The randomized, open-label, phase 3 study KEYNOTE-48 demonstrated the superiority of pembrolizumab monotherapy in PD-L1-positive patients and was not inferior compared to the standard first-line EXTREME (fluorouracil/platinum/cetuximab) regimen for R/M HNSCC. When replacing cetuximab (Erbitux^®^), a humanized monoclonal antibody targeting the epidermal growth factor receptor (EGFR), in the ternary combination with cisplatin and 5-fluorouracil (5-FU), pembrolizumab achieved better outcomes in all patient groups compared (11). Consequently, in 2019, the indication was extended and pembrolizumab has now been approved as first-line treatment for R/M HNSCC (11). Although several studies have confirmed the efficacy and safety of pembrolizumab in R/M HNSCC, they also showed that predicting response to PD-1 blockade remains challenging (9–14). To date, no definitive biomarker allows sufficient patient selection. PD-L1 expression is found in over 55% of HNSCC (15, 16) and is associated with better response to anti PD-1 antibodies such as pembrolizumab (4, 16). However, the subgroup of PD-L1-negative patients who also benefit from treatment with pembrolizumab emphasizes the need for complementary investigation (14).

Since the complex regulation of immune response is not limited only to the interaction of immune checkpoints, consideration of further mechanisms that influence immune activity might allow the prediction of response. Interferon gamma (IFN-γ) is a central coordinator of the innate and adaptive arms of the immune system. The biosynthesis of the cytokine by T lymphocytes, natural killer (NK) cells, and NK T cells leads to a TH1 cell-dominated microenvironment establishing antitumor effects, such as increased chemokine expression, enforcement of cytotoxic activity, upregulation of major histocompatibility complex (MHC) class I and II proteins, and inhibition of Tregs (17, 18). On the other hand, IFN-γ induces negative feedback inhibition by upregulating PD-L1 in cancer cells and compromises the immune response (19, 20). Analyses of the IFN-γ-related gene expression signatures indicated an association between the presence of the cytokine’s gene products and response to PD-1 blockade (21). Consequently, investigation of the diverse IFN-γ effects and their influence on the cytokine milieu interfering with successful immune checkpoint blockade (ICB) might add to response prediction. The value of IFN-γ as a prognostic biomarker has already been demonstrated. Lower IFN-γ concentrations are associated with nodal metastasis in HNSCC (22). However, because IFN-γ underlies a circadian regulation (23, 24), using it alone as a biomarker could lead to inconsistent results. IP-10 has already been proven to be a suitable, stable chemokine to monitor IFN-γ activity (25). IP-10 is a potent chemoattractant protein that is produced by a variety of cells, e.g., mononuclear cells, fibroblasts, and endothelial cells, upon stimulation with IFN-γ (26). It is able to reduce tumor growth, regulate angiogenesis, and increase the recruitment of cytolytic lymphocytes into tumor lesions (27, 28). Ayers et al. showed that tumors with high expressions of IFN-γ-related genes, including IP-10, reflect a response to pembrolizumab therapy better than IFN-γ alone (21).

Interleukin 6 (IL-6), a well-researched cytokine found in high serum concentrations in many cancer patients (29) and is also produced through cancer cells (30), is known to induce the secretion of other cytokines such as IL-8 (CXCL8), vascular endothelial growth factor (VEGF), and CCL2 (monocyte chemoattractant protein 1, MCP-1), altogether exerting pro-tumoral effects in an autocrine and paracrine manner (31).

With an incidence of approximately 800,000 newly registered cases per year, HNSCC is one of the top 10 malignancies worldwide (32). Despite great advances in surgery, radiotherapy, chemotherapy, targeted therapy, and, recently, the introduction of immune checkpoint inhibitors, poor prognosis and high mortality make further investigation and development of treatment strategies necessary (33).

To demonstrate not only the efficacy but also the effects of the synergism/additivity or even antagonism of pembrolizumab alone, or in combination with cetuximab and the effects of IFN-γ, an *ex vivo* assessment of tumor cell colony formation (CFec) was conducted by utilizing histopathologically confirmed HNSCC samples in the FLAVINO assay (34). We report on the cytokine profiles observed in supernatants and the association of varying responses to *ex vivo* treatment with the outcomes of HNSCC patients.

## Materials and Methods

### Patients and Pathological Tumor Data

The study NICEI-CIH, a prospective cohort study analyzing the neoantigen spectrum, immunogenicity, and clinical efficacy of immune checkpoint inhibitors in HNSCC, was approved by the Ethics Committee of the University Leipzig (ethic vote 341-15-05102015) and was conducted according to the guidelines of the Declaration of Helsinki. All patients provided written informed consent. A total of 23 patients with histologically confirmed HNSCC were included in the study. Tissue biopsies from each patient were acquired by ENT (ear, nose, and throat) surgeons during definitive surgery or panendoscopy at the ENT Clinic of the University Hospital Leipzig, Germany. The patients were treated according to the consented decision of the Multidisciplinary Tumor Board (MDTB). Detailed characteristics of the included patients are shown in **Table 1**.

**Table 1:** Characteristics of the HNSCC samples investigated in the FLAVINO assay.

Sex	Age	Localization	TNM 8th ed.^†^	UICC 8th ed^†^	p16 status	Grading
M	713	OPSCC	T1 N M0	I	positive	3
M	80.9	LHSCC	T4a N0 M0	IVA	negative	3
M	60.9	other	T4a N2c M1	IVC	negative	2
M	58.8	OPSCC	T1 N3b M0	IVB	negative	3
F	74.6	OPSCC	T3 N2 M0	II	positive	3
M	69.0	LHSCC	T4a N2b M0	IVA	negative	3
M	69.0	LHSCC	T4a N2b M0	IVA	negative	3
F	69.5	OPSCC	T4a N1 M0	III	positive	3
M	63.0	other	T4b N0 M0	III	unknown	4
M	68.9	OPSCC	T3 N3a M0	IVB	negative	2
M	65.2	OPSCC	T4a N3a M1	IVC	negative	3
M	72.3	OPSCC	T3 N2 M0	III	positive	2
M	73.0	OPSCC	T1 N2a M0	IVA	negative	2
F	59.6	OPSCC	T3 N1 M0	II	positive	3
M	59.3	OPSCC	T4a N2a M0	IVA	negative	2
M	58.0	OPSCC	T3 N1 M0	II	positive	2
F	62.8	LHSCC	T2 N2c M0	IVA	negative	3
M	63.4	LHSCC	T4a N3b M0	IVB	negative	3
M	56.6	OPSCC	T2 N1 M0	I	positive	3
M	78.9	OPSCC	T3 N3b M0	IVB	negative	2
M	71.5	OPSCC	T2 N0 M0	II	negative	2
F	53.5	LHSCC	T4a N0 M0	IVA	negative	3
M	56.4	LHSCC	T3 N0 M0	IVC	negative	2

^†^Stage was according to TNM staging for solid tumors, 8th edition (2017).

OPSCC, oropharyngeal squamous cell carcinoma; LHSCC, laryngeal/hypopharyngeal squamous cell carcinoma.

### FLAVINO Assay

The FLAVINO assay, a clonogenic, *ex vivo* chemoresponse evaluation test, was performed as described in previous publications (34–36). The culture medium used was phenol red- and flavin-free RPMI 1640 (Bio&Sell, Feucht, Germany) supplemented with 10% fetal calf serum (FCS; Anprotec, Bruckberg, Germany) and penicillin, streptomycin, amikacin, and nystatin C (all from Sigma-Aldrich, Deisenhofen, Germany) as antimicrobial agents. The experiments were performed under monochromatic light from sodium lamps (Philips, Hamburg, Germany) to avoid phototoxic reactions (37, 38). After overnight digestion of the previously mechanically disintegrated freshly obtained tissue with collagenase IV (Sigma-Aldrich, Deisenhofen, Germany), about 3 × 10^5^ viable cells/ml (final number, 3 × 10^4^ cells/well) were distributed and seeded into 96-well microtiter plates coated with collagen I, laminin, and fibronectin (Roche, Mannheim, Germany). The cell cultures were treated with either a single compound or combinations of 50 µg/ml cetuximab (Erbitux^®^; Merck Serono, Darmstadt, Germany) and 50 µg/ml pembrolizumab (MK-3475; Merck Sharp & Dohme Corp., Whitehouse Station, NJ, USA) alone or supplemented with 10 ng/ml IFN-γ (Peprotech GmbH, Hamburg, Germany). Twelve wells were left untreated and used as the control. After incubation for 72 h under standard culture conditions (36.5°C, 3.5% CO_2_, 95% humidity), culture supernatants were collected from all the cultures and stored at −80°C until cytokine measurements, applying indirect sandwich enzyme-linked immunosorbent assay (ELISA; see below). The adherent cells were then fixed by consecutively using 40% and 70% ethanol. After air drying and blocking nonspecific binding with an assay buffer containing 1% (*v*/*v*) FCS, this was followed by pan-cytokeratin detection utilizing a primary murine monoclonal antibody (SC 8018, C11; Santa Cruz Biotechnology, Santa Cruz, CA, USA) diluted 1:800 in phosphate-buffered saline (PBS) containing 0.5% FCS and 0.05% Tween-20™ and subsequent staining with fluorescein isothiocyanate (FITC)-labeled antibodies [goat-anti mouse immunoglobulin G (IgG) FITC] (#31569; Thermo Scientific, Rockford IL, USA). The colony formation of epithelial cells (CFec) was examined using immunofluorescence microscopy (inverted microscope Axiovert 200M; Carl Zeiss, Oberkochen, Germany). Fifteen of the 23 HNSCC samples showed adherent growth (mean CFec ≥ 4/well in 12 wells of sham-treated controls), and the mean of each individual untreated control used to normalize the colony formation was expressed as a percentage of the control.

### ELISA

The cytokine concentrations in cell-free culture supernatants were measured in indirect sandwich ELISA utilizing OptEIA™ kits (BD Biosciences, Heidelberg, Germany) for IL-6, IL-8, IP-10, MCP-1, and IFN-γ and VEGF-EDK kits for VEGF_165_ (#900-K10; Peprotech, Hamburg, Germany) according to the manufacturers’ instructions. Tetramethyl benzidine was used as a substrate. After measuring the optical densities at λ_1_ = 450 nm and λ_2_ = 620 nm on the Synergy2™ multi-mode microplate reader (BioTek Instruments, Inc., Winooski, VT, USA), we calculated the calibration curves using Gen5™ software (BioTek Instruments, Inc., Winooski, VT, USA). The lower limit of detection (LLD) was ≤2 pg/ml and the lower limit of quantification (LLQ) was ≤4 pg/ml for all cytokines.

### Drug Interactions

In order to objectively evaluate the interactions between the used drugs, we calculated the changes from baseline (untreated control) to achieve the delta values *P* expressed as rational numbers, further used to calculate the interaction measure *q* (34, 35, 38–43) applying the following formula (Eq. 1):


(1) q=P[A+B]/(P[A]+P[B]−P[A]×P[B])

where *P*[*A*] stands for the effect exerted by cetuximab, *P*[*B*] for the effect of pembrolizumab, and *P*[*A + B*] for the combination of both cetuximab and pembrolizumab. For instance, if cetuximab would have reduced colony formation to 80% of the control (by 20%), pembrolizumab reduced it to 90% (by 10%), and their combination to 65% (by 35%), based on Eq. 1, *q* will be 1.25:


q= 0.35/(0.2+0.1−(0.2×0.1))=0.35/0.28=1.25.

This *q* = 1.25 indicates synergistic suppression as, according to the model of independent action of drugs (34, 35, 38–43), *q*-values > 1.15 indicate synergistic effects, whereas *q-*values between 0.85 and 1.15 reflect additivity. Values below 0.85 indicate antagonism of treatments, i.e., pure antagonistic effects or effects significantly below independent action (39–41).

### Statistical Analysis

All data shown were based on 12 replicate well measurements for each treatment or control for each HNSCC patient. To assess differences between groups, the values obtained and values normalized to untreated controls were expressed as the mean and 95% confidence interval (95% CI) or the median and interquartile range (IQR; the distance between the 25th and 75th percentiles) and were compared applying Student’s *t*-test for heteroscedastic samples or the Mann–Whitney *U* test, respectively. Two-sided ANOVA was used to assess variance between treatments and patients. The distributions of categorical variables were compared using contingency tables and Pearson’s chi-square (*χ* ^2^) tests. Receiver operating characteristic (ROC) curves were applied to identify the optimum cutoff values for binary split of parameters according to the maximum Youden index (maximum product of specificity and sensitivity) for treatment failure (progressing disease or cancer-related death). Progression-free survival (PFS) was measured from the time of diagnosis (i.e., obtaining the sample analyzed in the FLAVINO assay) until detection of treatment failure, either progressing disease, relapse, or cancer-related death, censoring patients who are alive or those dying from other causes. Differences in the time-to-event data (PFS) were assessed using log-rank tests. Calculations and statistical analyses were performed using Excel 2016 (Microsoft Corporation, Redmond, WA, USA) and IBM SPSS Statistics for Windows, version 25.0. (IBM Corporation, Armonk, NY, USA), and *p*-values ≤0.05 in two-sided tests were regarded significant.

## Results

### Colony Formation

**Figure 1:**
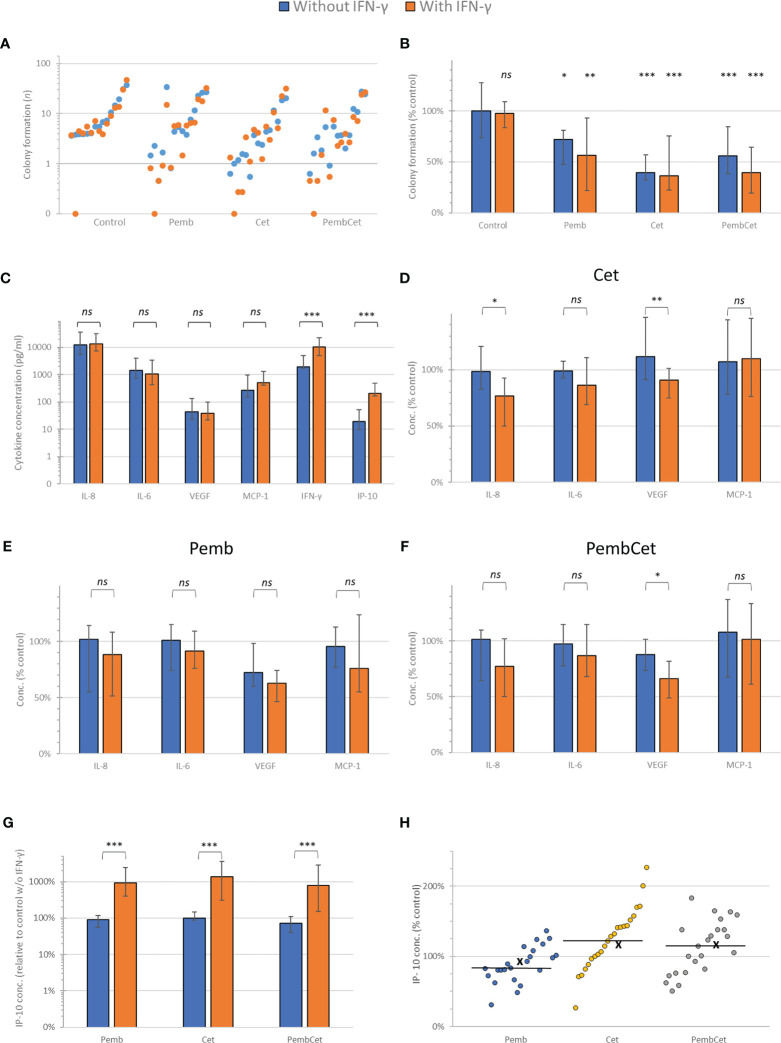
Efficacy of pembrolizumab (*Pemb*), cetuximab (*Cet*), and the combination of both antibodies (*PembCet*) without (*blue*) or with interferon-gamma (IFN-γ) in the FLAVINO assay. **(A)** Dot plot representing the mean number of epithelial cell colonies formed (CFec) by the individual head and neck squamous cell carcinoma (HNSCC) samples sorted in ascending order of CFec in 12 control replicates without IFN-γ and accompanied by the corresponding mean CFec under IFN-γ treatment. **(B)** Median and interquartile range of CFec normalized to the untreated control (100%) by Pemb, Cet, and PembCet without or with 10 ng/ml IFN-γ showing the huge heterogeneity of HNSCC in response to treatments. **(C)** Mean and 95% CI of the cytokine concentrations in supernatants of untreated controls and after IFN-γ stimulation at 72 h **(D–F)** Cytokine concentrations normalized to the untreated control without and with IFN-γ stimulation at 72-h treatment with cetuximab **(D)**, pembrolizumab **(E)**, and PembCet **(F)**. **(G)** Effect of treatment indicated on interferon gamma-induced protein 10 (IP-10) production normalized to the control without IFN-γ (please note logarithmic scaling). **(H)** Dot plot showing the IP-10 concentrations observed for individual HNSCC samples as percentage of individual IFN-γ-treated control (*x*, mean; *horizontal line*, median). *p < 0.05; **p < 0.005; ***p < 0.001; *ns*, not significant.

Of the 23 HNSCC samples analyzed, 15 (65.2%) had sufficient colony formation of epithelial cells (mean CFec ≥ 4/well), allowing assessment of the effects of pembrolizumab, cetuximab, and pembrolizumab + cetuximab and their combination with IFN-γ (**Figures 1A, B** **)**. Pembrolizumab, cetuximab, and their combination significantly reduced the tumor colony formation (**Figures 1A, B** **)**. Comparison of the CFec reduction with and without IFN-γ stimulation showed that a statistically significant difference was achieved through all treatments. In the otherwise untreated but IFN-γ-stimulated control, with the exception of only one HNSCC with complete loss of colony formation, no significant CFec reduction was shown. The mean colony formation (CF) in controls without IFN-γ was 10.8 colonies/well (95% CI = 6.6–15) compared to 10.5 colonies/well (95% CI = 5.6–15.4) with IFN-γ (*p* = 0.939). ANOVA revealed a significant impact of the various antibody treatments on CFec, exceeding the variance in untreated controls independent of using number of colonies per well (*p* = 0.0051) or after the normalization of CFec to the individual patient’s mean value observed in the untreated control (*p* = 1.719 × 10^−06^). More specifically, pembrolizumab treatment with or without IFN-γ reduced the CFec by about 44% and 28%, respectively (*p* < 0.01 and *p* < 0.05, respectively). Cetuximab without IFN-γ reduced the CFec by 61.5% and after stimulation by 63.3% (both *p* < 0.001). The binary combination suppressed the CFec by 54% without and 61.5% with IFN-γ stimulation (both *p* < 0.001), demonstrating a boost on the average pembrolizumab effect through cetuximab. However, the differences between the mean and 95% CI values demonstrated the highest variability in CFec in response to pembrolizumab, and an excess in CFec observed in a few HNSCC cases led to a mean above that of the control, whereas the median and the 75th percentile were below (**Figure 1B**). The addition of IFN-γ reduced the adverse (stimulating) effects of pembrolizumab and led to a reduced heterogeneity, according to the smaller 95% CI and IQR, an effect that was also achieved through combining pembrolizumab and cetuximab. However, after Bonferroni correction for multiple testing, only cetuximab or cetuximab together with pembrolizumab remained significant CFec suppressors.

### Effects of IFN-γ on Colony Formation Following Pembrolizumab and Cetuximab

In 80% (12/15) of the samples, we observed antagonistic effects between pembrolizumab and cetuximab. Additivity was found in three samples (3/15, 20%), and no synergistic effect was registered. The addition of IFN-γ drastically changed the interactions of both antibodies. In more than half of the HNSCC cases (8/15, 53.3%), additive effects were observed, whereas in 2/15 (13.3%) pembrolizumab and cetuximab worked synergistically. Antagonism (effects below additivity) was found in only 5 of 15 (33.3%) HNSCC cases.

Interestingly, in 12 of 15 samples responding to pembrolizumab and cetuximab with antagonism, the addition of IFN-γ switched the mode of action of the antibody combination in 7 of 12 HNSCC cases. This was a switch from antagonism into synergism in 2 out of 12 samples and from antagonism into additivity in 5 out of 12 HNSCC cases. Furthermore, we found no switch from additivity or synergism to antagonism in the other three samples.

### Cytokine Release

Supernatants from all 23 HNSCC samples were acquired, and the cytokine release up to 72 h (IL-8, IL-6, VEGF, MCP-1, IFN-γ, and IP-10) was measured. We compared the changes in the production of each cytokine with and without the addition of IFN-γ (**Figures 1C–F**). With VEGF as the only exception, IFN-γ did not cause any significant reduction of cytokine concentrations (percent of the control). When treated with pembrolizumab, IFN-γ did not significantly reduce the cytokine concentrations (percent of the control) compared to HNSCC patients without IFN-γ treatment. In the cetuximab-treated group, IFN-γ suppressed, statistically significantly, the median concentrations of IL-8 and VEGF (*p* < 0.05 and *p* < 0.005, respectively) compared to those without IFN-γ treatment. Under the influence of IFN-γ, the combination of both antibodies led to a significant suppression of VEGF production compared to untreated samples.

While treatment with IFN-γ (10 ng/ml) increased the IP-10 production by about 10-fold, the concentrations of IP-10 measured in solely pembrolizumab- or cetuximab-treated wells demonstrated no deviation, and the combination of both antibodies only produced a slight reduction in IP-10 compared to the untreated control (all *p* > 0.1; **Figure 1G**).

In IFN-γ-stimulated HNSCC cultures, after treatment with pembrolizumab, we observed a huge heterogeneity in HNSCC, with most (15/23) samples showing reduced IP-10 concentrations and only in 8 of the 23 samples did the IP-10 concentrations increase compared to the IFN-γ-treated control (**Figure 1H**). In contrast, cetuximab increased IP-10 production in 17 and 15 out of 23 IFN-γ-stimulated HNSCC samples without and with pembrolizumab, respectively. This cetuximab effect was reflected by the mean and median IP-10 concentrations above those of the IFN-γ-treated control (**Figure 1H**).

### Interaction of Cetuximab and Pembrolizumab in Their Impact on Cytokine Release

**Figure 2:**
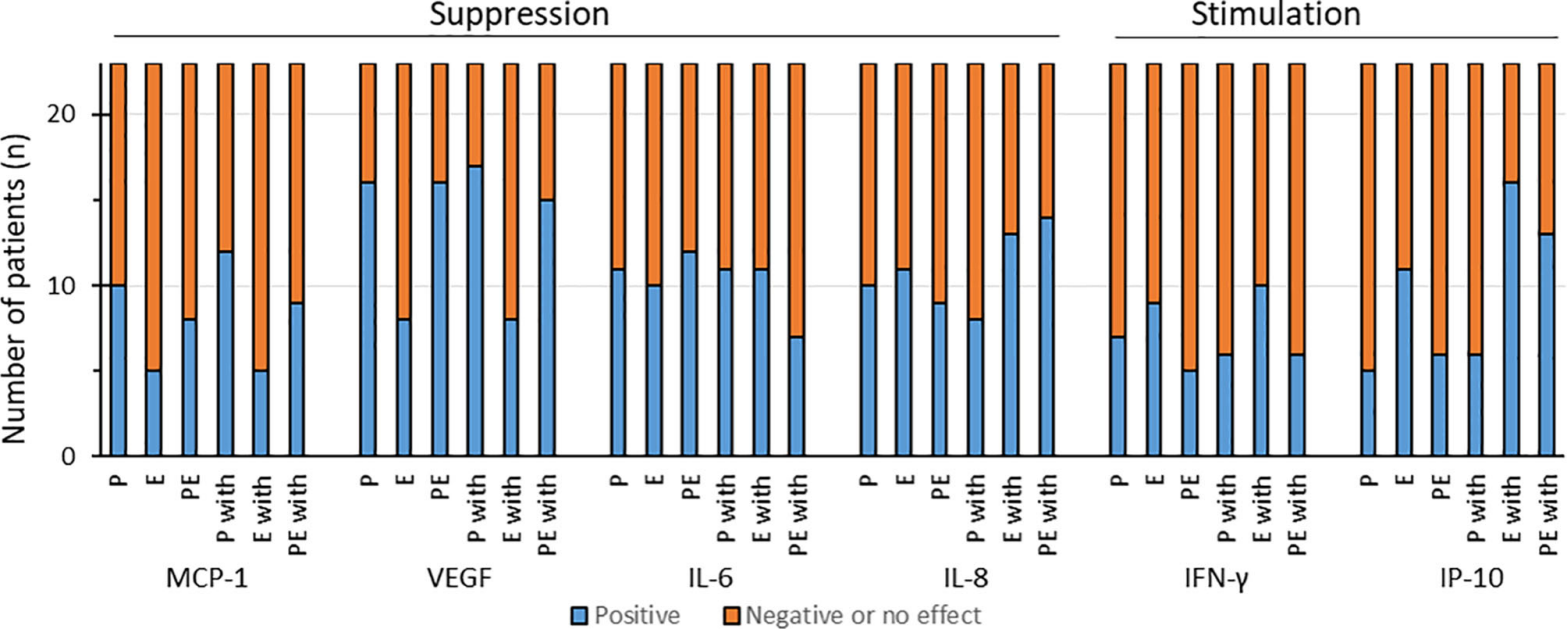
Distribution of the effects of pembrolizumab (*P*), cetuximab (*E*), and pembrolizumab plus cetuximab (*PE*) alone or with IFN-γ (*P with*, *E with*, or *PE with*, respectively) on the cytokine production of individual HNSCC samples. We defined as positive effect either the significantly suppressed production of monocyte chemoattractant protein 1 (MCP-1), vascular endothelial growth factor (VEGF), interleukin 6 (IL-6), and IL-8 or the significant increased production of interferon gamma (IFN-γ) and interferon gamma-induced protein 10 (IP-10) through synergism or additivity.

To assess the interactions of pembrolizumab and cetuximab, we examined and identified the overall effects regarding cytokine release after calculating the *q*-values, as described. A suppression through additivity or antagonism was considered as a positive effect for IL-6, IL-8, VEGF, and MCP-1, but a negative effect for IFN-γ and IP-10. According to the literature, for the latter two cytokines, stimulation was defined as a positive effect (**Figure 2**).

A positive effect on MCP-1 production was observed in less than 50% of the samples through pembrolizumab, cetuximab, and their combination (10/23, 5/23, and 8/23, respectively). The addition of IFN-γ changed the effect slightly when the HNSCC samples were treated with pembrolizumab alone (12/23 showed positive effects). Cetuximab and cetuximab plus pembrolizumab combined with IFN-γ demonstrated positive effects in 5 and 9 out of 23 samples, respectively (**Figure 2**).

In terms of VEGF secretion, the majority of the HNSCC cases treated with either pembrolizumab or pembrolizumab plus cetuximab (both 16/23) demonstrated positive effects, whereas such effects were observed in only 8 of the 23 samples treated with cetuximab alone. The addition of IFN-γ did not change much, as 17 of 23 after pembrolizumab and 15 of 23 after combination treatment delivered the desired effects. Cetuximab alone did not change anything in this regard (**Figure 2**).

Without IFN-γ stimulation, the suppression of IL-6 production, which could be interpreted as a beneficial response of HNSCC to treatment, was displayed by 11, 10, and 12 out of 23 samples (for pembrolizumab, cetuximab, and pembrolizumab plus cetuximab, respectively). The extra IFN-γ treatment, however, impaired the positive reaction to the binary treatment (pembrolizumab plus cetuximab) in five HNSCC samples, reducing it to only 7 out of 23 (**Figure 2**). Not much difference was shown through pembrolizumab and cetuximab alone (both 11/23 showing reduced IL-6).

Examination of IL-8 revealed that pembrolizumab, cetuximab, and pembrolizumab plus cetuximab affected cytokine secretion in a similar manner without substantial interaction, whereas the addition of IFN-γ to the binary combination reduced the release of IL-8 in the majority of samples (**Figure 2**). We registered lowered IL-8 concentrations, which we interpreted as a beneficial effect, in 10, 11, and 9 out of 23 samples. IFN-γ clearly improved the response to pembrolizumab plus cetuximab, as now 14 out of 23 HNSCC samples reacted positively (lowered IL-8 concentrations). An improvement was also seen after cetuximab treatment (13/23), whereas fewer samples demonstrated this effect when treated with pembrolizumab alone (8/23).

Interestingly, we observed positive effects only in the minority of HNSCC patients when taking IFN-γ into consideration, i.e., with and without the addition of IFN-γ. More specifically, after pembrolizumab treatment, only 7 out of 23 showed positive effects; for cetuximab and pembrolizumab plus cetuximab, these numbers were 9 and 5 out of 23, respectively. Similar results were found after IFN-γ stimulation, where pembrolizumab and pembrolizumab plus cetuximab led to positive effects in only 6 out of 23 and cetuximab in 10 out of 23 HNSCC cases.

IP-10 production was stimulated through cetuximab alone in 11 of 23 HNSCC patients. Only 5 and 6 out of 23 samples displayed similar results with pembrolizumab and the combination of both antibodies. Simultaneous stimulation with IFN-γ significantly increased IP-10 through cetuximab treatment. A heightened IP-10 production was observed in 13 out of 23 HNSCC samples when treated with cetuximab and pembrolizumab plus IFN-γ, which led to IP-10 levels exceeding those induced by IFN-γ treatment alone. Almost no increase in IP-10 concentrations above those induced by IFN-γ was seen in HNSCC samples treated with pembrolizumab plus IFN-γ, as an increased IP-10 release was registered only in 6 out of 23 HNSCC samples.

### IP-10 Score, a Prognostic Biomarker in HNSCC

**Figure 3:**
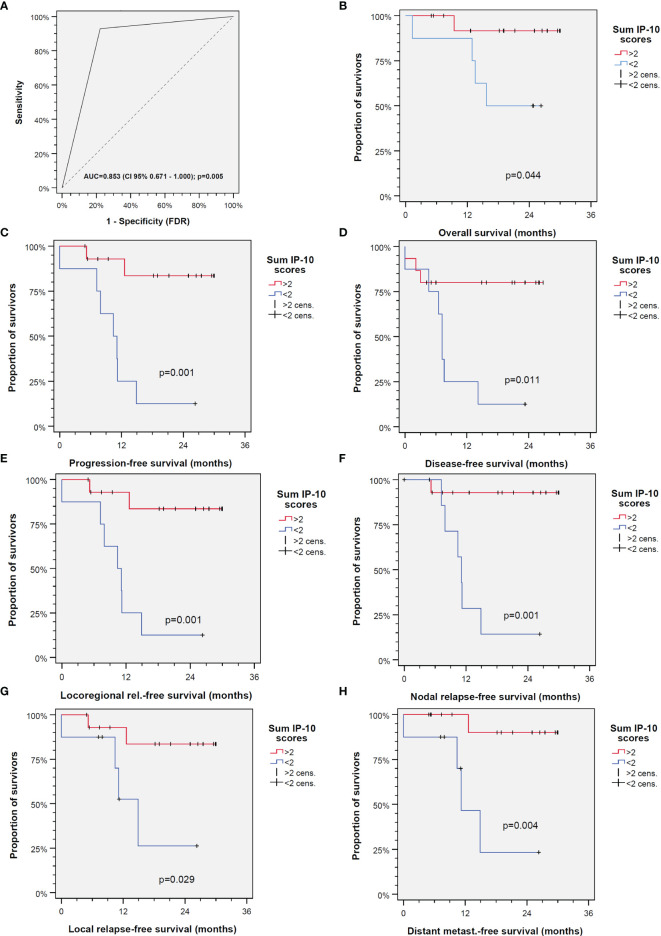
Interferon gamma-induced protein 10 (IP-10) is able to predict outcome of head and neck squamous cell carcinoma (HNSCC) independent of the treatment applied. **(A)** Receiver operating characteristic (ROC) curve for the IP-10 score (*AUC*, area under the curve). **(B–H)** Outcomes of patients with IP-10 score >2 (*red line*) *versus* ≤2 (*blue line*) for overall survival **(B)**, progression-free survival **(C)**, disease-free survival **(D)**, locoregional relapse-free survival **(E)**, nodal relapse-free survival **(F)**, local relapse-free survival **(G)**, and distant metastasis-free survival **(H)**. The *p*-values shown are from two-sided log-rank tests.

We investigated individually the IP-10 production in all the tested samples. Utilizing ROC curves, we identified the cutoff values for every patient for all drug combinations with and without the application of IFN-γ (**Figure 3**). We assigned one point for every treatment category if the value was over the cutoff. Values below the cutoff were not assigned any points. We then summed up the points of every patient, yielding the IP-10 score, and again used the ROC curves to define its cutoff value. We found that this point system offers relevant prognostic information not only about the general course of the disease but also about specific events such as the probability of relapse. More specifically, as shown in **Figure 3**, patients with an IP-10 score over 2 have a significantly prolonged overall survival (OS; *p* = 0.044), PFS (*p* = 0.001), disease-free survival (*p* = 0.0011), locoregional relapse-free survival (*p* = 0.001), nodal relapse-free survival (*p* = 0.001), local relapse-free survival (*p* = 0.029), and distant metastasis-free survival (*p* = 0.004).

## Discussion

In our series of experiments, we demonstrated the effects of IFN-γ on CFec and the cytokine profiles of HNSCC cell cultures under treatment with pembrolizumab, cetuximab, and the combination of both antibodies utilizing the FLAVINO assay. The FLAVINO assay is a non-clonogenic short-time chemoresponse assay developed and patented in our laboratory (US provisional ser. no. 61/044,082). It allows the *ex vivo* examination of cell cultures of epithelial tumors such as HNSCC (34, 42). Non-clonogenic assays such as FLAVINO are superior to clonogenic assays when studying heterogeneous tumors such as HNSCC as they allow a more accurate assessment of the tumor behavior by differentiating cancerous epithelial and stromal cells and also release soluble proteins, including cytokines and growth factors, related to the response to treatment (43).

Although the introduction of monoclonal antibodies against PD-1 such as pembrolizumab has revolutionized the therapy against many cancer forms, including HNSCC (8), and it is now being used as a first-line agent against R/M HNSCC after positive results from the KEYNOTE-048 study (11), only a fraction of patients show long-term response under this treatment (44, 45). A major factor leading to this phenomenon is the ability of the tumors to compromise the immune response in the tumor microenvironment (TME) and gain resistance to immune checkpoint inhibitors (46), mainly through insufficient T-cell infiltration of the tumor, T-cell exhaustion, and also the lack of response to IFN-γ (44, 47), which are important anticancer mechanisms used by the immune system. The heterogeneity of many tumors such as HNSCC (36) is also believed to be a factor leading to acquired resistance to immunotherapy (48).

The anti-EGFR monoclonal antibody cetuximab has been used for many years as first-line or second-line systemic treatment for metastatic and/or recurrent HNSCC alone or in combination with other antineoplastic agents, e.g., cisplatin chemotherapy and 5-FU (EXTREME regimen) (49) or taxane instead of 5-FU (TPExtreme) (50).

Studies revealed that cetuximab enhances the antitumor immune response of the innate immune system through the activation of NK cells in HNSCC and, more importantly, of the adaptive immune response through facilitating the impact of NK cells *via* their FcγRIII (CD16) on tumor cells and also involves dendritic cells (DCs), which leads to T-cell antitumor immunity, a crucial part of the overall complex antitumor response (51, 52). As NK cells express not only CD16 (stimulating antibody-dependent cell-mediated cytotoxicity, ADCC) but also PD-1 (suppressing ADCC), combining cetuximab and pembrolizumab may overcome cetuximab resistance. Moreover, a subpopulation of patients receiving cetuximab responds to murine peptides of the variable (murine) region of cetuximab. This means that epithelial cells will, following digestion of the EGFR:cetuximab complexes in their lysosomes to peptides, present those including murine peptides to cytotoxic CD8^+^ T cells, and their effectiveness is also reduced in the presence of PD-L1. Therefore, the effects through cetuximab and pembrolizumab and *vice versa* could establish optimal conditions in the TME for both anti-EGFR and anti-PD-1 antibodies to achieve better and long-term responses (53).

Indeed, we were able to demonstrate the beneficial effects of cetuximab and pembrolizumab in the majority of the samples, as increased IFN-γ-concentrations were measured. This can be interpreted as an indirect sign of the reactivation of exhausted T cells and/or increased proliferation of tumor-infiltrating lymphocytes. Pembrolizumab treatment with and without cetuximab led to a significantly reduced median CFec. In addition, the single-agent treatment with cetuximab significantly reduced the CFec. With the exception of only one HNSCC sample responding to IFN-γ with a completely abolished colony formation, IFN-γ alone did not show significant effects on the CFec, probably because the tumor-infiltrating lymphocytes were already in an inactive state through interaction with checkpoint molecules presented by tumor cells. IFN-γ may have facilitated antigen processing and presentation in the single HNSCC case, but otherwise seems to have limited impact on cytokine production, except on IP-10.

The beneficial effects of cetuximab in HNSCC could also be observed on the CFec in our *ex vivo* experiments. With the exception of only one HNSCC sample responding to IFN-γ with a completely abolished colony formation, the addition of IFN-γ to the antibodies did not show significant improved effects on CFec compared to unstimulated samples. Similar results were also seen in the samples treated with pembrolizumab and cetuximab, where a significant CFec reduction was found, but no substantial (significant) gain was noticed in the presence of IFN-γ stimulation.

However, based on the results from *ex vivo* and *in vivo* studies in mice (54–56), we expected a substantial increase in antitumor activity following the addition of IFN-γ, reflected by the reduced IL-6 and VEGF production and the diminished CFec based on facilitated ADCC and activity of cytotoxic T cells. In these studies (54–56), *in vitro* using 4-nitroquinolone-1-oxide neoplastic transformed squamous cells were used to establish cell lines that either rejected (regressors) or grew progressively (progressors) when transplanted into immune competent mice (54). While regressors expressed high levels of B7.1 (CD80), progressors did not (54), and the dichotomy in CD80 expression was found to be critical for response to systemic IL-12 and peritumoral IL-2 immunotherapy, as only the tumors grown from CD80^+^ cell lines responded. The response, however, was abrogated in IFN-γ-deficient mice, whereas CD80 expression could be restored by IFN-γ treatment. In contrast, the NF-κB-dependent cytokines IL-1, IL-6, and GM-CSF suppressed CD80 expression in the progressor cell lines (56). In light of these observations, the increase of IFN-γ simultaneous to the reduced IL-6, VEGF, and CFec, and even the more pronounced IP-10 release and reduced IL-6, VEGF, and CFec after IFN-γ stimulation after treatment with both monoclonal antibodies demonstrated in our *ex vivo* series of experiments, could be interpreted as promising. Indeed, pembrolizumab combined with cetuximab was able to significantly reduce the CFec and also showed a reduction of the VEGF concentrations, which was even stronger after IFN-γ-stimulation.

A combination therapy for HNSCC is currently under investigation, but is yet to become a treatment option. A recent phase II study in which 33 HNSCC patients received treatment with pembrolizumab and cetuximab delivered promising results with good response rates and low rates of adverse events (57).

In our experiments, the combination of both antibodies increased the IP-10 concentrations in some HNSCC samples that did not respond to pembrolizumab with IP-10 release, suggesting a compensation of the anti-IP-10 effects of pembrolizumab in these HNSCC samples. These findings support the indirect hypothesis that a combination of both monoclonal antibodies could be a potent alternative to the existing therapies for R/M HNSCC potentially able to overcome an immunosuppressive cancer micro-milieu, a hurdle to overcome during anticancer therapies.

Interactions between drugs when used together are inevitable. We showed in previous publications that synergistic or additive effects are possible, but an antagonistic mode of action leading to the opposite rather than desired results can also occur (39, 58). This unpredictability of the mode of action was also seen in this study. In terms of the cytokine profiles, all the above results make obvious that the effects not only of the combination of pembrolizumab and cetuximab but also of the two antibodies used alone are very heterogeneous. Although some consistent results were observed, no specific tendency that appears to be generalizable was found in this context. Nevertheless, this is no surprise when studying highly heterogeneous tumors such as HNSCC.

Developing a test that is able to predict the outcomes of a malignant disease has always been challenging, especially because multiple factors influence the outcome (35, 36). In heterogeneous cancers like HNSCC, a reliable prediction is even more difficult as, sometimes, even samples of the same tumor show different behaviors in various tests (36). However, we identified remarkable differences between the samples in their IP-10 response to pembrolizumab, cetuximab, and the combination of both to the added IFN-γ. Mostly, IFN-γ induced 10-fold IP-10 levels, but the further stimulation of IP-10 release by at least two out of the three antibody treatments was found to be prognostic for a significant superior outcome (**Figure 3**). The IP-10 score described here precisely predicts the outcome of the 23 HNSCC patients studied. It predicted differences in not only OS but also PFS, disease recurrence, and metastatic events. More importantly, this IP-10 score can predict the previously mentioned events independently, requiring only IP-10 concentrations from the short-time FLAVINO assay without adjustments for potentially interfering factors. Further investigations, however, are needed to prove the value of IP-10 measurements in *ex vivo* cultures for therapy decision-making.

## Conclusion

Because of this heterogeneity, it appears extremely difficult to precisely predict the outcome of the disease in patients with HNSCC only by measurement of the available biomarkers. In our series of experiments, we demonstrated the importance of IFN-γ and IP-10 as biomarkers in the treatment of HNSCC. We developed a score based on IP-10 secretion that allows predicting the course of the disease in HNSCC utilizing the FLAVINO assay independent of the treatment applied. Not only PFS and OS can be predicted, but also more specific events such as relapse probability and metastasis-free survival. This demonstrates the relevance of an appropriately functioning immune response for outcomes in HNSCC that can reliably be assessed using the FLAVINO assay.

## Data Availability Statement

The raw data supporting the conclusions of this article will be made available by the authors, without undue reservation.

## Ethics Statement

The studies involving human participants were reviewed and approved by The Institutional Human Ethics Committee of the University Leipzig. The patients/participants provided their written informed consent to participate in this study.

## Author Contributions

Conceptualization: GW. Methodology: GW. Validation: MB, IM, SW, and GW. Formal analysis: MB, IM, SW, and GW. Investigation: MB, IM, and GW. Resources: AD, VZ, MP, SW, and GW. Data curation: MB, IM, and GW. Writing—original draft preparation: MB, IM, and GW. Writing—review and editing: all authors. Visualization: MB, IM, and GW. Supervision: SW and GW. Project administration: GW. Funding acquisition: GW and AD. All authors contributed to the article and approved the submitted version.

## Conflict of Interest

AD and SW received honoraria for invited lectures from MSD. AD received funding from MSD for an investigator-initiated clinical trial (ADRISK, ClinicalTrials.gov, no. NCT03480672).

The remaining authors declare that the research was conducted in the absence of any commercial or financial relationships that could be construed as a potential conflict of interest.

## Publisher’s Note

All claims expressed in this article are solely those of the authors and do not necessarily represent those of their affiliated organizations, or those of the publisher, the editors and the reviewers. Any product that may be evaluated in this article, or claim that may be made by its manufacturer, is not guaranteed or endorsed by the publisher.
